# A Retrospective Case Study into the Effect of Hoof Lesions on the Lying Behaviour of Holstein–Friesian in a Loose-Housed System

**DOI:** 10.3390/ani11041120

**Published:** 2021-04-14

**Authors:** Karen Jiewei Ji, Richard E. Booth, Nicola Blackie

**Affiliations:** Pathobiology and Population Sciences, Animal Welfare Science and Ethics, Hawkshead Campus, Royal Veterinary College, Hawkshead Lane, Hatfield, Hertfordshire AL9 7TA, UK; kji6@rvc.ac.uk (K.J.J.); rbooth@rvc.ac.uk (R.E.B.)

**Keywords:** dairy cow, lameness, sole ulcer, digital dermatitis, lying time, mobility score, IceQube

## Abstract

**Simple Summary:**

Lameness is a substantial welfare and economic problem in production animals. It can alter indicators of welfare such as lying time. Lying down is very important for cows, and they are highly motivated to perform this behaviour for 12 h or more per day. Conversely, cows that lie down too much or are uncomfortable standing may miss an opportunity to feed or drink if there is competition from sound (non-lame) cows. This study monitored different lesions that cause lameness in cattle through the use of accelerometers. The lesions included sole ulcers, sole haemorrhage, white line disease, interdigital hyperplasia and phelgmon, and digital dermatitis. Leg-based activity monitors that track the cows’ lying behaviour and mobility were used. From these data, it was found that cows with lesions on the foot spent longer lying down than those with no lesions, and cows with lesions in the soft tissue spent less time lying down than those with foot lesions. Trimming the cows’ feet altered the lying times of the cows with foot lesions and returned them closer to those of cows with no lesions.

**Abstract:**

The association between hoof lesions and lying behaviour was assessed on a Holstein–Friesian dairy farm in England. Twenty-nine cows were included in the study. Cows with claw horn disruption lesions (CHDL, *n* = 8), soft tissue lesions (STL, *n* = 6), and no lesions (NL, *n* = 15) were assessed. Data were collected on parity, days in milk (DIM), and mobility scores. Cows were trimmed and treated, and lesions were recorded by a professional foot trimmer. Lying behaviour was assessed before and after claw trimming. The milking herd (*n* = 96) prevalence of lameness was 32.3%. Mobility was scored using the Agriculture and Horticulture Development Board (AHDB) Mobility Scoring system. Mobility scores were significantly different across lesions groups (*p* = 0.022). CHDL cows had a mean mobility score of 2.0 ± 0.9 (mean ± SD), STL were scored 1.2 ± 1.3, and NL cows were 0.9 ± 0.7. CHDL were associated with longer lying times (15.00 ± 1.04 h/d; *p* = 0.0006) and shorter standing times (9.68 ± 2.38 h/d; *p* = 0.0351) compared with NL lying times (11.77 ± 1.67 h/d) and standing times (12.21 ± 1.67 h/d). STL cows spent significantly less time lying (11.30 ± 2.44; *p* = 0.0013) than CHDL but not NL cows. No significant differences were found with any of the other lying behaviours. After trimming, CHDL cows spent significantly less time lying down than before trimming (13.66 ± 0.98; *p* = 0.0125). Cows with NL spent significantly more time lying down (12.57 ± 1.90; *p* = 0.0398) and had a shorter minimum lying bout duration (0.17 ± 0.09; *p* = 0.0236) after trimming. In conclusion, lying behaviour in dairy cattle was impacted by type of hoof lesions and hoof trimming.

## 1. Introduction

Lameness in dairy herds poses significant economic and welfare problems [1,2]. Furthermore, a consumer concern has led to lameness detection being included in various farm assurance schemes. Estimates of lameness prevalence vary with a range of 20.6–36.8% [1,3,4,5,6,7]; however, this varies globally due to different management systems. Recent publications suggest UK dairy cow lameness prevalence to be 30.1% to 31.6% [3,6], indicating that lameness is a significant issue in UK dairy farming.

Lameness in dairy cows is most commonly associated with the hindlimb [4,8], with over 90% of lesions found on the foot [5]. These can be divided into two broad categories: soft tissue lesions (STL), which may have an infectious component, and claw horn disruption lesions (CHDL) caused by trauma or increased pressure. Soft tissue lesions include digital dermatitis (DD), interdigital hyperplasia (IH), and interdigital phlegmon. Claw horn disruption lesions include sole ulcers (SU), sole hemorrhage (SH), and white line disease (WLD). Sole ulcers, WLD, and DD are the most common lesions affecting UK and Irish dairy herds [5,9]. Foot lesions are painful; however, as a prey species, cows are stoic in nature, and so, they often do not show signs of pain until lesions are advanced [10,11]. Foot lesions and lameness as well as being painful are related to decreased reproductive performance [12,13,14], reduced milk yield [15,16], and an increased likelihood of culling [17,18]. A drop in milk yield can be seen up to 4 months before diagnoses/treatment and up to 5 months after, resulting in an average of 350 kg of milk lost/cow/lactation [19]. Similarly, a significant drop in milk yield has been reported up to 3 months before treatment, suggesting that pathogenesis begins far before lameness is seen [20]. Furthermore, lesion-specific decreases in yield have been found with estimates of 570 kg and 370 kg loss due to SU and DD, respectively [20].

In addition to productivity, lameness affects the behaviour of dairy cattle, particularly feeding and lying behaviour. Several confounding factors influence these behaviours including different management and housing styles [21,22,23,24] and environmental variables [25,26,27]. Behavioural changes are associated with cow-level factors such as days in milk (DIM), parity, and body condition score (BCS) [16,28,29]. Lame animals feed less frequently [30] and often after non-lame cows [31]. They also have altered lying behaviour; higher locomotion score cows have longer lying times with fewer longer lying bouts [22,29,32,33,34]. Specific hoof lesions, such as claw horn disruption lesions (CHDL), have been repeatedly reported to have shorter standing times [16] and increased lying times [35,36]. After claw trimming, SU and DD cows lie less than healthy control groups [37]. The literature is unclear about the effects of lesions on lying bout frequency; CHDL are reported to have both numerically fewer bouts [36,38] and more bouts [16] than non-lame cows. The importance of lying time in dairy cows is related to welfare, health, and reproductive status [39,40]. Cows are highly motivated to lie down, spending 9–14 h/d resting, prioritising lying over feeding, and other social behaviours [41]. Cows may have a behavioural requirement to lie down for 12–13 h/d [42].

The prompt detection and treatment of lame animals is essential, as animals rarely self cure, and treatment delays are inevitably associated with increased severity [43]. Several methods of lameness detection have been described including ad hoc observation, locomotion scoring, and routine hoof trimming. Ad hoc observation is ineffective with mild/moderate cases and translates poorly when recording herd statistics [1]. Farmers in the UK have underestimated lameness prevalence, failing to identify three out of four cases [44]. Serial locomotion scoring is highly recommended [45], although scoring is subjective. Locomotion scores have been associated with foot lesions [46,47,48]; however, not all severe lesions result in obvious lameness [11]. Forty percent of severe foot lesions were locomotion scored as 2 or 3 out of 5, which were described as imperfect and mildly abnormal locomotion, respectively [11]. Ideally, most cows should be trimmed 2–3 times per year [48]. Foot trimming alone may see lesions going unnoticed for prolonged periods as sole lesions can take anywhere from 6 to 8–10 weeks to appear at the sole surface.

Automated lying time measurements may be a useful adjunct to lameness detection for farmers [49]; they may aid in early detection and treatment, thus improving welfare [43] and limiting production losses [15]. It is suggested that one accelerometer per cow is most useful in a cost–benefit analysis [50]. Investments in these systems are usually justifiable, with 84% of cost–benefit scenarios breaking even within the system’s 10-year lifespan [51]. Accelerometers do not influence dairy cow lying behaviour; thus, they can give accurate herd statistics [52,53].

While the effects of lameness on lying behaviour are well documented, few papers assess specific hoof lesions and their effects on behaviour. This study aims to explore the effects of specific hoof lesions on the lying times of Holstein-dairy cows using the CowAlert system within a loose housed system.

## 2. Materials and Methods

### 2.1. Ethical Approval

This study was approved by the Clinical Research Ethical Review Board (CRERB) at the Royal Veterinary College, London; reference number CR2020-052-2.

### 2.2. Animals and Management

The study was performed on a 112-cow dairy farm in Hertfordshire, UK. This number represents milking animals and cows that have been dried off. Milking Holstein–Friesian cows (*n* = 96) were identified for the study, and 80 were randomly fitted with activity monitors by the herdsperson in the milking parlour. Data were collected from January to March 2020. There were 13 primiparous cows and 55 multiparous cows who had activity monitor data available (parity = 3.61 ± 1.99; mean ± SD (standard deviation)). Cows were loose-housed indoors on a majority woodchip bedding mixed with a recycled gypsum plasterboard product. The flooring in the yard was grooved concrete. The collecting yard, parlour, and raceway had rubber matting. The floors were scraped twice daily. High yielders were fed a mixed ration consisting of grass and maize silage, straw, brewers’ grains, and a mineral blend in addition to concentrates in the parlour (fed according to yield). The ration was presented at 6:00 every morning and pushed up every hour by a robotic feed pusher. Minimal concentrates were given in the parlour to low yielders. High yielders were milked twice daily at 5:45 am and 3:00 pm while low yielders were milked once at 8:00 am.

### 2.3. Data Collection

**Figure 1:**
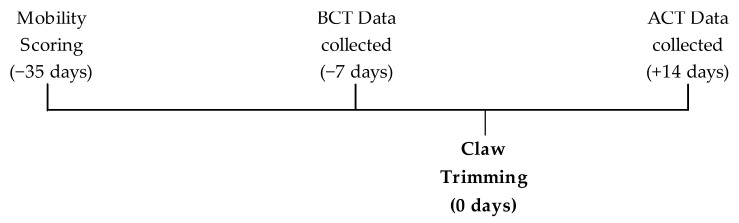
Timeline of key events within the study.

Leg-based activity monitors (Cow Alert; IceQube, Ice Robotics LTD., Edinburgh, UK) were fitted with a Velcro-strap above the hindlimb fetlock in mid-February 2020. The activity monitors were left on after the study was completed to be included as a part of the management system. The cows recruited for this study had a minimum of 72 h to adapt to the activity monitors, which is within the described ranges previously used for habituation [52,54,55]. The monitors collected data on lying behaviour, activity with 4 Hz 3-dimensional accelerometers, several times per second. The average data for lying behaviour over 7 days was used 1 week before claw trimming (BCT) and 2 weeks after claw trimming (ACT). Claw trimming was performed in early March. The time frame was selected based on the literature, which suggests that lying behaviour may be altered 1 week before [56] and 2–3 weeks after claw trimming [37]. A timeline of key events is shown in Figure 1.

Mobility scores were assessed for the milking herd (*n* = 96) using the Agriculture and Horticulture Development Board (AHDB) dairy mobility scoring system [57] (Table 1). This was completed at the end of January 2020, 5 weeks BCT. This timeline was chosen as SU can take up to 6 weeks to appear at the sole surface. The assessors were trained in mobility scoring. This was undertaken following afternoon milking as the cows were walking out of the milking parlour on concrete. Cows with a MS ≥ 2 were considered lame.

The farm trims every cow at least once a year prior to dry off and also treats lame cows as they are picked up by ad hoc observation. A professional foot trimmer visited the herd in March. Cows with SU were trimmed, treated with a block applied to the unaffected claw, and given Ketoprofen. DD cases were treated twice daily for three days with oxytetracycline spray in the milking parlour. The Ketoprofen and oxytetracycline treatments were performed by the herdsperson as per standard foot care protocol. In addition, all cows were regularly footbathed (up to 6 times/week) with 4% formalin as a preventative measure. Foot trimming records for 63 cows were collected.

Following trimming, the data from the cows were grouped into 3 categories. The first included those with soft tissue and/or infectious lesions (STL), which included DD and interdigital hyperplasia (IH). The second group consisted of those with CHDL, which encompassed SU, sole hemorrhage (SH), sole separation, laminitis and WLD, and cows with no lesions (NL). The last group included cows with uncommon lesions (*n* = 6); which included forelimb lameness (*n* = 4) and cull cows (due to lameness). Those with simultaneous STL/CHDL (*n* = 2) were excluded. For the purposes of this study, IH and DD were grouped together as STL, based on the strong associations for these conditions in the literature [32,58,59]. Twenty-two cows did not have complete data from the sensors and so were excluded. This left 29 cows in the final analyses.

### 2.4. Statistical Analysis

The 7-day average (mean) for lying behaviour (Table 2) from the IceQubes was analysed before claw trimming (BCT) and after trimming (ACT). The 7-day average was pre-calculated by the activity monitors. Lying behaviour was recorded in hours and minutes and converted into hours/day for statistical analysis.

Prism 8 (GraphPad) software was used for most of the analyses; R 4.0.0 (The R Foundation; Vienna, Austria) was used to analyse mobility scores, as this calculation could not be completed with Prism 8. For each lying behaviour measurement analysed, normality was assessed with a D’Agostino and Pearson skewness test (CHDL and NL) and a Shapiro–Wilk test (STL). Results with normal distributions, BCT (lying time, min and max lying bout, lying bouts/day), and ACT (standing time, lying bouts/day) were analysed with a one-way ANOVA. If significant, a Tukey’s multiple comparison was performed. All other datasets, BCT (standing time), and ACT (lying time, min and max lying bout) were analysed with a non-parametric Kruskal–Wallis test. The prevalence of mobility scores was analysed with a Fisher’s exact test. The effects of hoof lesions on lying times BCT and ACT were analysed using a paired *t*-test for normally distributed data, and skewed data were analysed with a Wilcoxon matched pairs test. The effect of hoof lesions on minimum lying bout duration BCT and ACT were analysed with a Wilcoxon matched pairs test, as all data were skewed. A *p*-value of <0.05 was considered statistically significant.

## 3. Results

### 3.1. Lameness and Lesion Prevalence

The milking herd prevalence of lameness was 32.3% as determined by the MS of 96 cows. Foot trimming records were available for 63 cows, of which 33 had one or more foot lesions present. Of these, 28.6% (18/63) of cows had one affected foot, 20.6% (13/63) had two affected, and 4.7% (2/63) had ≥3 affected. The most prevalent foot lesions were DD, IH, SU, and SH, from most to least common, respectively (Table 3). Twelve sensors failed prior to data analysis. After grouping the applicable cows (cows with foot trimming records and activity monitor data), the data from a total of 29 cows were analysed; 8 CHDL, 6 STL, and 15 NL cows.

### 3.2. Mobility Scores

Herd prevalence was based on 96 milking cows (out of 112, including dry cows), and the results are as follows: 27.1% were MS-0 (*n* = 26), 40.6% were MS-1 (*n* = 39), 24% were MS-2 (*n* = 23), and 8.3% were MS-3 (*n* = 8). There were no cows with MS-0 among those that had CHDL, while NL cows had no MS-3 cows. Mobility scores were significantly different between the specific lesion groups with CHDL score 2.0 ± 0.9; STL score 1.2 ± 1.3 and NL score 0.9 ± 0.7 (*p* = 0.022).

### 3.3. Parity and Days in Milk (DIM)

Neither parity nor DIM were statistically significant between lesion groups. CHDL had a numerically higher parity, 4.6 ± 2.6 (mean ± SD), while NL (3.1 ± 1.5) and STL (3.5 ± 2.1) had average parities slightly above 3. The mean DIM for all groups indicated mid-late lactation stage at trimming. NL cows had the highest mean DIM (246.0 ± 104.3), followed by CHDL (209.3 ± 79.0) and STL (172.3 ± 101.1).

### 3.4. Before Claw Trimming (BCT)

Lying behaviour did vary between lesion groups (Table 4). CHDL spent significantly more time lying than the NL group. There was no significant difference in lying time between cows with STL and NL. CHDL cows spent an additional 3.2 h and 3.7 h lying compared to NL and STL cows, respectively. CHDL spent significantly less time standing than NL cows. While minimum and maximum lying bout duration did not vary significantly, CHDL cows did tend to have longer maximum lying bouts (*p* = 0.085) and numerically more lying bouts on average.

### 3.5. After Claw Trimming (ACT)

Lying and standing time did not vary significantly between lesion types (Table 4). Number and duration of lying bouts did not differ significantly between groups.

### 3.6. Comparison between BCT and ACT

**Figure 2:**
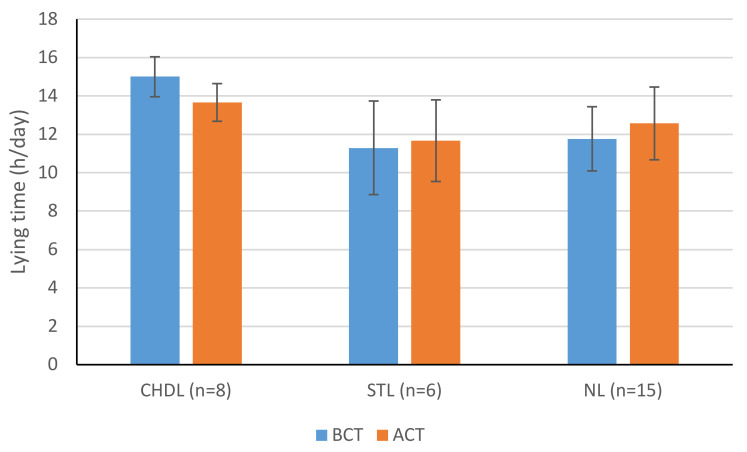
Analyses of the effects of hoof lesions on lying time before (BCT) and after claw trimming (ACT) in 29 Holstein–Friesian cows. Lying times for each lesion were assessed on two different occasions (BCT and ACT) to determine if there was a significant difference (*p* < 0.05). Normally distributed data was analysed with a paired *t*-test; * Skewed data was analysed with Wilcoxon matched pairs test.

**Figure 3:**
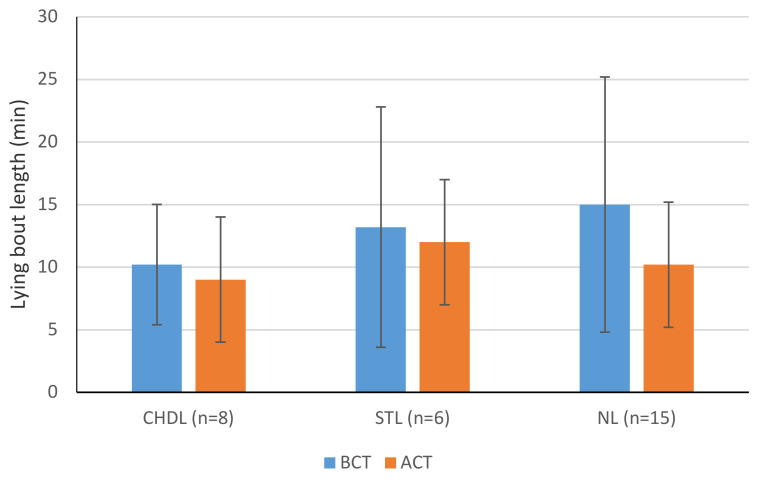
Analyses of the effect of hoof lesions on minimum lying bout duration before (BCT) and after claw trimming (ACT) in 29 Holstein–Friesian cows. The minimum lying bout duration for each lesion was assessed on two different occasions (BCT and ACT) to determine if there was a significant difference (*p* < 0.05). All data were skewed and analysed with a Wilcoxon matched pairs test.

Lying time decreased significantly from BCT to ACT for CHDL cows (15.00 vs. 13.66 h/d; *p* = 0.0125) (Figure 2). STL and NL groups increased their lying time, although the change was significant only for NL cows (11.77 vs. 12.57 h/d; *p* = 0.0125). Cows with NL also had significantly shorter minimum lying bout duration ACT (Figure 3). No other lying behaviours were statistically significant when comparing BCT and ACT.

## 4. Discussion

The herd prevalence of lameness was 32.3%, which is similar to other studies into lameness prevalence in the UK [3,6,7]. The majority of lesions (92%) were found on the hindlimbs, which is in line with reports from other studies [5,60]. In general, lying times in the present study complement other studies. Lying times for cows with NL or STL ranged from 11.3 to 12.6 h/d, others have quoted cows lying down for 12 h/d in free-stall systems [61] and 10.5–13.6 h/d in loose-housed systems as per the current study housing [62,63,64]. The cows in the present study, especially post-trimming, fulfill the 12–13 h/d lying behavioural need [42]. Before claw trimming, CHDL cows were found to lie significantly longer than both STL and NL; however, STL do not lie significantly less than NL cows.

It has been repeatedly shown that lame cows lie longer than non-lame cows, coupled with shorter standing times, due to their inverse association [33,65,66]. In contrast, shorter lying times have also been associated with lame animals [37,49,61], and increased standing times are often implicated as a risk factor for lesion development [61,67]. Before trimming, cows with CHDL laid down for an additional 3.3 h/d versus NL and 3.7 h/d versus STL cows. This is longer than other papers [33], and the variance could be due to a number of factors such as of housing design [22], lesion severity, or cow level factors such as BCS, parity, and stage of lactation [29]. When assessing cow-level factors in this study, it was found that neither parity nor DIM varied significantly between lesion groups.

Individual cows show great variation in lying times within a given farm [68]. Chapinal et al. found that SU cows spent 1.1 h/d longer lying than non-lame cows [36]; this was not true for SH or DD cows in their study. CHDL cows in the present study spent an additional 3.23 h/d lying versus NL cows. Theoretically, CHDL share a common aetiology where contusions within the claw horn capsule cause sole lesions [69]. Cows may be trying to relieve pressure on CHDL to alleviate the associated pain while, anatomically, DD lesions are not directly impacted by weight bearing when standing. Lying behaviour was only an indicator of STL, mainly DD, in another study [70]. Navarro et al. found lame cows, with sole damage and infectious lesions, spent less time standing (13.5 h/d) than non-lame cows (15.2 h/d) [16]. The effects of IH alone may be limited but are variable based on lesion size and severity and whether there are any concurrent infectious/traumatic lesions associated.

Lying bout duration seems to increase in higher MS cows [22,33]. In particular, cows with sole damage have longer lying bouts [16,71]. The literature has also found that longer lying bout durations were associated with STL, but not CHDL, specifically [70]. Lying bouts/day can vary; generally, studies have found lame cows have fewer, longer lying bouts [22,32,33], with more variation in lying bout length [21]. In this study, CHDL may have numerically more lying bouts/day, which is unusual, although they do demonstrate a wider range of lying bout length. Lame cows have been reported to have more lying bouts/day; however, this may be attributed to differences in automatic milking systems [72]. In this study, all three groups did not vary significantly in lying bout length or bouts/day.

The difference in lying behaviour BCT and ACT was significant for CHDL and NL cows. After trimming, CHDL cows spent less time lying and NL cows spent more time lying. This may indicate increased comfort in CHDL cows post-treatment. Other studies have found similar effects where cows with SU and DD spent more time standing 2–3 weeks ACT [37]. While no significant changes were seen with STL cows, this may be attributed to the mild effects of IH lesions on the cows in the group. Cows have been seen to increase daily lying time in the period after trimming, this was found in lame and non-lame cows [56,71]. Thus, cows with NL may be more tender after trimming. Conversely, in another study, cows with a foot-block laid down longer than non-lame cows ACT, but no other treatment group saw any change in lying behaviour [73]. This included those given a block and nonsteroidal anti-inflammatory drugs (NSAID) [73]. Pain associated with the lesions is the likely cause; investigations on the effects of the blocks themselves have been shown to increase MS, they do not seem to alter lying times when applied to non-lame cows [74]. A decrease in the minimum lying-bout duration of NL cows was significant post-trimming (*p* = 0.0236). This may indicate greater comfort in walking and transitioning from a lying position.

Mobility score was significantly different across the lesion groups in this study. CHDL had the highest mean MS, while STL and NL cows were often not classified as lame. The indication that MS may be more useful for identifying CHDL than STL has been documented previously [70,75]. Sole ulcer [47,48], double sole, and interdigital purulent inflammation [46] are associated with increased locomotion scores. Sole hemorrhage [47], WLD, and DD [46] have not been noted to change MS. When WLD is assessed with SU as CHDL, there seems to be an association with MS [76], as seen in this study. DD is a painful condition; however, its association with lameness is more variable [36,46]. It may be related to chronicity [11] or severity [77] wherein acute lesions would be expected to cause pain. In addition, due to the location of DD, the cow should not apply direct pressure with the lesion when weight bearing. It has also been found that DD cows did not appear lame unless concurrent CHDL was present [78]. Likewise, IH has a lesser association with lameness [79]. In general, the current study agrees with the literature in that CHDL, especially SU, are associated with visible lameness. STL do seem to be associated with a slightly higher MS than for cows with NL; however, the location, stage, and severity of these lesions was not noted in this study. Further categorisation of these lesions with a bigger sample size may have yielded different results.

Study limitations included sample size and the inability to check mobility scores at trimming and after trimming. Although parity and DIM were not statistically significant between groups, analysing cows with a particular stage of lactation and parity may yield more specific results. BCS was not assessed, although it is associated with lameness [80,81], CHDL [69,82], and lying times [29]. Cows were assessed in broad lesion groups as opposed to specific lesions categorised according to location and severity.

In summary, lying times may be a useful adjunct for lameness detection to mobility scoring and regular foot trimming. Cows with CHDL lie for significantly longer periods than other cows. This extreme behaviour may be used to identify cows that require further examination, although more work is needed to determine what changes deem further investigation necessary. Monitors may also be useful to monitor the efficacy of treatment, CHDL spent less time lying, making them more comparable to cows with NL than pre-trimming, possibly indicating a greater index of comfort. The use of activity monitors is already widespread due to their benefits for heat detection, so implementing their use for lameness detection is quite feasible. Future work looking at the effect of specific foot lesions on lying behaviour would be interesting and may prove to be more useful for detecting lameness.

## 5. Conclusions

Mobility score and increased lying times or decreased standing times can be used as indicators of CHDL in dairy cows. While not a perfect means of identifying lesions, they can be used as tools for farmers to identify cows that may require attention. The benefits of hoof trimming can also be seen up to two weeks ACT. Cows with CHDL and NL showed beneficial changes after treatment.

## Figures and Tables

**Table 1:** Agriculture and Horticulture Development Board (AHDB) dairy mobility scoring system [57].

Category	Score	Description of Behaviour
Good mobility	0	Walks with even weight bearing and rhythm on all four feet, with a flat back. Long, fluid strides possible.
Imperfect mobility	1	Steps uneven (rhythm or weight bearing) or strides shortened; affected limb or limbs not immediately identifiable.
Impaired mobility	2	Uneven weight bearing on a limb that is immediately identifiable and/or obviously shortened strides (usually with an arch to the centre of the back).
Severely impaired mobility	3	Unable to walk as fast as a brisk human pace (cannot keep up with the healthy herd). Lame leg easy to identify—limping; may barely stand on lame leg/s; back arched when standing and walking. Very lame.

**Table 2:** IceQube (IceRobotics) accelerometer monitoring descriptions of lying behaviour.

Measure	Description
Lying time	Time in hours (h) that the sensor is positioned horizontally.
Standing time	Time in hours (h) that the sensor is positioned vertically.
Lying bout length	Period between the sensor changing from vertical to horizontal, then back to vertical.
Step count	The number of times the cow lifts her leg, based on the amount of force used.

**Table 3:** Distribution of hoof lesions (number (%) by foot per cow) from 63 cows in a Holstein–Friesian dairy herd.

Leg	SH	SU	Laminitis	WLD	Sole Separation	DD	IH	Other *
Front:								
Left	0 (0%)	0 (0%)	1 (1.5%)	0 (0%)	0 (0%)	0 (0%)	0 (0%)	0 (0%)
Right	1 (1.5%)	0 (0%)	0 (0%)	1 (1.5%)	1 (1.5%)	0 (0%)	0 (0%)	1 (1.5%)
Hind:								
Left	4 (6.3%)	3 (5%)	1 (1.5%)	0 (0%)	1 (1.5%)	7 (11.1%)	5 (8%)	7 (11.1%)
Right	1 (1.5%)	5 (8%)	1 (1.5%)	1 (1.5%)	1 (1.5%)	9 (14.2%)	11 (17.5%)	2 (3.2%)

Sum of the percentages may not equal 100% due to rounding. SH = solar haemorrhage, SU = sole ulcers, WLD = white line disease, DD = digital dermatitis, IH = interdigital hyperplasia. * Other includes wall ulcers, toe necrosis, fissures, abscesses, toe/leg damage.

**Table 4:** Effect of hoof lesions on lying behaviour (lying and standing time, lying bout duration and frequency) before (BCT) and after claw trimming (ACT) for 29 Holstein–Friesian cows.

	CHDL (*n* = 8)	STL (*n* = 6)	NL (*n* = 15)	*p*-Value
**Lying time (h/d)** **BCT**	15.00 ± 1.04 ^a,c^	11.30 ± 2.44 ^b^	11.77 ± 1.67 ^b,x^	0.003
Standing time (h/d) * **BCT**	9.68 ± 2.38 ^a^	12.69 ± 2.43 ^a,b^	12.21 ±1.67 ^b^	0.021
**Min lying bout (min)** **BCT**	10.2 ± 4.8	13.20 ± 9.6	15.0 ± 10.2 ^x^	0.483
**Max lying bout (min)** **BCT**	169.2 ± 21.0	141.0 ± 21.0	147.0 ± 30	0.085
**Lying bouts/day** **BCT**	11.8 ± 2.3	10.0 ± 2.2	10.0 ± 2.2	0.193
**Lying time (h/d)** ACT *	13.66 ± 0.98 ^d^	11.67 ± 2.12	12.57 ± 1.90 ^y^	0.176
**Standing time (h/d)** **ACT**	10.28 ± 0.97	12.32 ± 2.11	11.32 ± 1.68	0.083
Min lying bout (min) * **ACT**	9.0 ± 4.8	12.0 ± 9.0	10.2 ± 5.4 ^y^	0.878
Max lying bout (min) * **ACT**	177.6 ± 24.6	142.8 ± 33	165 ± 43.8	0.126
**Lying bouts/day** **ACT**	11.3 ± 0.7	11.0 ± 2.8	10.9 ± 1.9	0.927

Datasets analysed by one-way ANOVA. ^a,b^ within a row indicates significant differences found by a Tukey’s multiple comparison between lesion groups. * analysed with non-parametric Kruskal–Wallis. ^c,d^ within a column indicates significant differences found by a paired *t*-test. ^x,y^ within a column indicates significant differences found by a Wilcoxon matched pairs test. Mean ± standard deviation (SD) represented in each lesion group column.

## Data Availability

The datasets used and analysed during the current study are available from the corresponding author on reasonable request.
